# 

**Published:** 1893

**Authors:** 


### LITERARY.

A CHAPTER ON CHOLERA FOR LAY READERS; History, Symptoms, Prevention and Treatment of the Disease.

By Walter Vought, Ph. B., M. D., Medical Director and Physician in charge of the Fire Island Quarantine Station, Port of New York; Fellow of the New York Academy of Medicine. Illustrated with colored plates and wood engravings. In one small 12mo volume; 110 pages. Price 75 cents net. Philadelphia, The F. O. Davis Co., publishers, 1914 and 1916, Cherry st. It is an admirable treatise on the subject.

The September ATLANTIC MONTHLY contains an article which will be of special interest for a considerable number of readers, on "Edwin Booth," by Mr. Henry A. Clapp, the eminent Boston critic, whose appreciation of Mr. Booth is equal to his skill and literary art in adequately describing him as an actor. A second article of special value just now is one on "Wildcat Banking in the Teens," in which the historian J. Bach McMaster gives much information with regard to the old state banks which some people fear are to be restored. Bradford Torrey contributes an article, in his particular line of outdoor interest, on "The St. Augustine Road." Charles Egbert Craddock continues with even increased vigor the serial story "His Vanished Star." Charles Stewart Davison has an article of very great interest on Swiss travel, "A Slip on the Ortler." Agnes Repplier writes in her incisive and engaging way on "A Kitten." "A Russian Summer Resort" furnishes a theme of excellent variety for Miss Isabel F. Hapgood. Miss Preston and Miss Dodge continue their translations and notes on Petrarch's Correspondence. Sir Edward Strachey, who has furnished several attractive articles lately, contributes a paper on "Love and Marriage."

Houghton, Mifflin & Co., Boston.

The REVIEW OF REVIEWS for September is a number of fine variety and timeliness. It epitomizes and synchronizes the whole planet for the month of August, 1893. It discusses the monetary crisis, the silver debate, the tariff outlook, the Bering Sea decision, the French attack on Siam, the progress of the Home Rule bill, the politics of the European continent, various matters at Chicago and the World's Fair, and a hundred other timely subjects, the whole number being profusely illustrated with portraits and pictures. A sketch of Engineer

Ferris and his great wheel is a singularly readable and attractive article, and Mr. Stead contributes a most noteworthy character sketch of Lady Henry Somerset. There is an illustrated review of the fascinating story of Joan of Arc, the inspired Maid of Orleans, and a group of papers on the silver question by professors in the University of Chicago. The "Leading Articles of the Month" are notably well selected, while the "Record of Current Events" gives one a summary day by day of the remarkable course of the recent monetary crisis, and the carton reproductions in the "Current History in Caricature" are uncommonly entertaining.

*The Century* has just come in possession of one of the most unique and important historical documents of the age. It is a record of the daily life of Napoleon Bonaparte on board the English ship which bore him into captivity at St. Helena, as contained in the hitherto unpublished journal of the secretary of the admiral in charge. The reports of many conversations held by the admiral with the deposed Emperor regarding his important campaigns are given with great fullness, and there is much about the bearing and the personal habits of Bonaparte during the voyage. The Memoirs of Las Casas contain the story of the Emperor's deportation, as told by a Frenchman and a follower; this diary is an English gentleman's view of the same memorable journey, and of the impressions made by daily contact with the man who had had all Europe at his feet.

The diary will be published in early numbers of *The Century.*

As an illustration of the money paid to writers as soon as they acquire a reputation, the September COSMOPOLITAN contains less than eight thousand words, for which the sum of sixteen hundred and sixty-six dollars was paid. Ex-President Harrison, Mark Twain and William Dean Howells are the three whose work commands such a price.

The September number has more than one hundred illustrations, giving the chief points of interest in the Columbian Exposition, and the Fair is treated by more than a dozen authors, including the famous English novelist, Walter Besant; the Midway Plaisance, by Julian Hawthorne; Electricity, by Murat HalsteaD; the Liberal Arts Building, by Kunz, the famous gem expert of Tiffany & Company; the Department of Mines, by the chief of that department, etc.

A feature of this number is a story by Mark Twain, entitled "Is He living or Is He Dead?"

The October number of the DELINEATOR, which is called the Autumn number, is an exceedingly attractive publication, displaying a splendid variety of the accepted styles for the season, and practical information on a wide range of topics interesting to women. Two special pattern articles have been prepared in addition to the usual monthly issue; one on Fitting out the Family for Autumn and Winter, and the other on Empire Gowns and Lounging Robes.

The World's Fair series is brought to a close by a paper on Children at the Fair, and a description of the various State Buildings and their uses. Many other attractive features grace its pages.

Banks & Brothers, of Albany, have just issued a Manual on Boards of Health and Health Officers, by Lewis Bolch, M. D., Ph. D., Secretary of the State Board of Health.

The Manual is a valuable and handy publication for members of local Boards of Health, health officers and all others interested in health matters. It is a practical working manual. It is almost indispensable to the profession. Price $1.50. Delivered upon receipt of price.

LIPPINCOTT'S for September contains a complete novel, for which departure the magazine is becoming famous, entitled "A Bachelor's Bridal," by Mrs. H. Lovett Cameron. A chapter is devoted to "The Cross-roads Ghost," of the series of Lippincott's notable stories. "Uncle Sam in the Fair," is clearly presented by Charles King, U. S. A. C. H. Rockwell, U. S. N., writes a bright tale on "A Sea Episode."

The number is good throughout.

No publication is more authoritative upon questions of the day than the POPULAR SCIENCE MONTHLY. Each month brings discussions of practical interest, and consequently proves valuable. The September number opens with a paper by Professor F. W. Toussig, on "Why silver ceases to be money." Reformatory Prisons and Lombroso's Theories, is interestingly treated by Miss Helen Zimmern. Miss M. O. Boland discusses "Scientific Cooking," and in a way most interesting.

The whole number is replete.

Several of the subscriptions for exposition stock were from $50,000 to $100,000, and several hundreds from $10,000 to $25,000. The people of Chicago subscribed as they had never subscribed before, nearly all good and substantial citizens contributing according to their means, so that never perhaps in the history of the world was so large a subscription made so readily and promptly.—*The Book of the Fair, by Hubert H. Bancroft.*

MCCLURE'S MAGAZINE for September is one of the most interesting periodicals of the month. Although only four numbers of the magazine have been issued, its fresh variety of entertaining articles and delightful stories, and its plentiful and interesting illustrations already takes rank with the best of our monthly periodicals.

FOES IN AMBUSH, by Capt. Charles King; J. B. Lippincott Company, Philadelphia, is one of those breezy stirring stories of military life in that wild region that lies between the old states and the Pacific slope. It is a typical story of frontier life.

GODEY for September is one of the best that finds its way to any reviewer's table. Distinctly a lady's magazine.

THE BLUE AND GRAY proves interesting as usual. The September number is an improvement in every particular over any previous issues.

